# Primary Solitary Osseous Hodgkin's Lymphoma: A Case Report and Review of the Literature

**Published:** 2012-05-30

**Authors:** B Geramizadeh, M Farzaneh, M Ramzi, O R Momenzadeh

**Affiliations:** 1 Transplant Research Center, Shiraz University of Medical Sciences, Shiraz, Iran; 2 Department of Pathology, Shiraz University of Medical Sciences, Shiraz, Iran; 3 Hematology Research Center, Shiraz University of Medical Sciences, Shiraz, Iran; 4 Department of Internal Medicine, Shiraz University of Medical Sciences, Shiraz, Iran; 5 Department of Orthopedic Surgery, Shiraz University of Medical Sciences, Shiraz, Iran

**Keywords:** Hodgkin's Lymphoma, Primary, Scapula

Dear Editor,

Herein we report a case of primary Hodgkin's lymphoma (HL) of the scapula presented with shoulder pain. A 24-year-old young and healthy man presented with left shoulder pain and swelling. There was no evidence of weight loss, sweating, fever or any systemic symptoms. Laboratory evaluation was normal except for mild leukocytosis.

C-reactive protein was negative and erythrocyte sedimentation was normal. Physical examination showed mild shoulder limitation of motion secondary to pain and swelling due to ill-defined mass. No peripheral lymphadenopathy was detected despite of thorough examination of the whole body. Plain X-ray showed an osteolytic lesion in the scapula. Magnetic resonance imaging (MRI) showed diffuse abnormal signal in the scapula and the coracoid process. There were soft tissue masses on both sides of spine of scapula indicating origin from the bone. Head of humerus was unremarkable (Figure 1). With the radiologic differential diagnoses of neoplastic process, the patient underwent open surgery to obtain an excisional biopsy. Pathologic examination of the biopsy showed diffuse infiltration of polymorphic population of hematopoietic cells composed of eosinophils, plasma cells, lymphocytes, PMN leukocytes and foamy histiocytes. There were many isolated mono and binucleated large Reed-Sternberg cells which were reactive with CD15, CD30 and Pax-5, but nonreactive with LCA, EMA and ALK (Figure 2 and Figure 3a and Figure 3b).

**Fig. 1:**
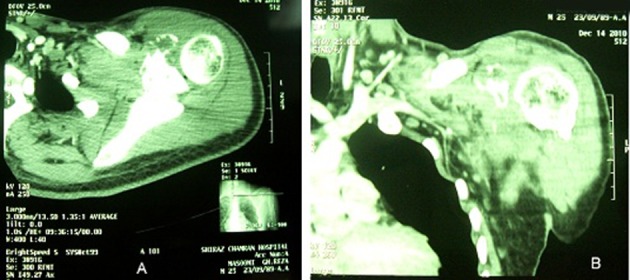
(A) CT scan of the scapula, (B) MRI of the scapula

**Fig. 2:**
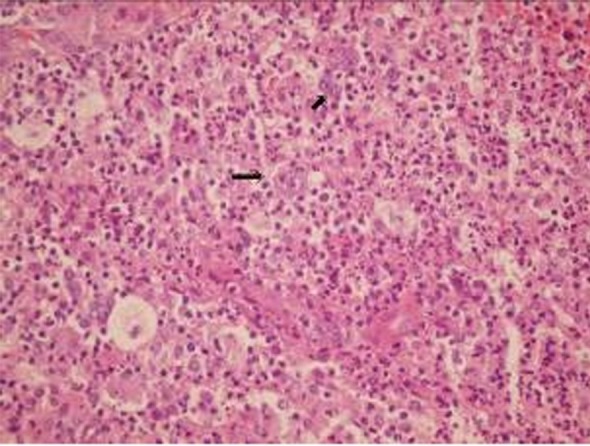
H and E section of the tumor (X250)

**Fig. 3:**
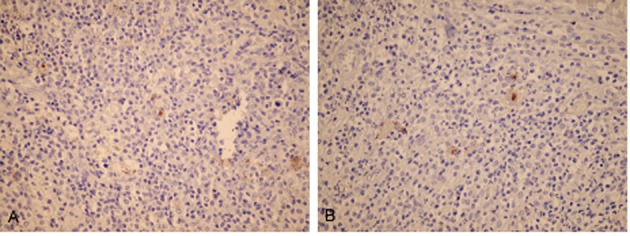
(A) IHC shows positive CD15 in RS cells, (B) IHC shows positive CD30 in RS cells

The pathologic diagnosis of classic HL, mixed cellularity was made. Abdominal and chest CT scans were re-evaluated but there was no sign of lymphadenopathy. Whole body scan showed increased radiotracer activity in the left scapula. No other focal abnormal uptake was seen in the rest of body.

Chemotherapy was started for him composed of doxorubicin (25 mg/m^2^), bleomycin (375 mg/m^2^), vincristine (6 mg/m^2^) and dacarbazine (375 mg/m^2^) given on days 1 and 15 repeated every 4 weeks. After 2 cycles of this therapy, the mass was completely disappeared and after 6 months, he was doing well and disease-free.

HL is a systemic disease with most common presentation in the lymph node.[1][2][3] Ten percent of HLs originate from extra nodal regions, but primary extra nodal presentation of HL occurs in less than 0.25% of patients.[4]

Bone involvement occurs in 10-20% of cases. Skeletal involvement may present in four different ways: Primary osseous HL (either solitary or multi focal), simultaneously in osseous and non-osseous sites, or recurrence of disease at osseous sites.[5] There are very few case reports of primary solitary osseous HL.[5][6][7] Most malignant lymphomas of bone are diffuse large B cell non-Hodgkin and in one study during 69 years (1927-1996) among 422 malignant lymphomas of bone, 13 cases of HL of bone were reported, but none of them has been primary solitary osseous HL.[7] To the best of our knowledge, less than 30 patients have been reported in the English literature with primary manifestation of HL in a single bone.[5][8] Mean age of the reported patients with solitary osseous HL has been about 35 years and most commonly involved bones were in the humerus, vertebra, iliac crest and femur.[5]

Our case was a 24-year-old man who presented with scapular pain and swelling. Scapula as a single bone of osseous HL has also very rarely been reported.[5]

The patient's general condition was very well and there was no clinical or radiologic evidence of HL involvement in other sites of the body. Treatment of the patient with ABVD was very successful and after 6 months, he was doing well and completely symptom-free. As a conclusion, HL can occur in the bone as the primary site and should be kept in mind in lytic lesions of scapula.

## References

[1] Cagavi F, Kalayci M, Tekin IO, Numanoglu G, Cagavi Z, Gül S, Açikgöz B (2006). Primary spinal extranodal Hodgkin's disease at two levels.. Clin Neurol Neurosurg.

[2] Madan M, Arora R, Singh J (2010). Solitary skeletal lesion as the primary manifestation of HL: a case report.. Acta Cytol.

[3] Bhagavathi S, Fu K (2009). Primary bone lymphoma.. Arch PAthol Lab Med.

[4] Samadian M, Vahidi S, Khormaee F, Ashraf H (2009). Isolated primary spinal epidural Hogdkin's disease in a child.. Pediatr Neurol.

[5] Langley CR, Garrett SJW, Uran J, Kohler J, Clarke NM (2008). Primary multifocal osseous Hodgkin's lymphoma.. World J Surg Oncol.

[6] Gebert C, Hardes J, Ahrens H, Buerger H, Winkelmann W, Gosheger G (2005). Primary multifocal osseous Hodgkin disease: a case report and review of literature.. J Cancer Res Clin Oncol.

[7] Ostrowski ML, Inwards CY, Strickler JG, Witzig TE, Wenger DE, Unni KK (1999). Osseous Hodgkin Disease.. Cancer.

[8] Oshikawa G, Arai A, Sasaki K, Ichinohasama R, Miura O (2009). Primary multifocal osseous Hogkin lymphoma.. Rinsho Ketsueki.

